# *Plasmodium falciparum*: Adhesion Phenotype of Infected Erythrocytes Using Classical and Mini-Column Cytoadherence Techniques

**Published:** 2013

**Authors:** N Kalantari, S Ghaffari, M Bayani

**Affiliations:** 1 Cellular and Molecular Biology Research Center, Babol University of Medical Sciences, Babol, Iran; 2 Dept. of Parasitology and Mycology, School of Medicine, Babol University of Medical Sciences, Babol, Iran; 3 Infectious and Tropical Disease Research Center, Babol University of Medical Sciences, Babol, Iran

**Keywords:** CD36, Cytoadherence, E-selectin, ICAM-1, *P. falciparum*, V-CAM

## Abstract

**Background:**

Cytoadherence of *Plasmodium falciparum*- infected erythrocytes to host cells is an important trait for parasite survival and has a major role in pathology of malaria disease. Infections with *P. falciparum* usually consist of several subpopulations of parasites with different adhesive properties. This study aimed to compare relative sizes of various binding subpopulations of different *P. falciparum* isolates. It also investigated the adhesive phenotype of a laboratory *P*. *falciparum* line, A4, using different binding techniques.

**Methods:**

Seven different *P*. *falciparum* isolates (ITG, A4, 3D7 and four field isolates) were cultivated to late trophozoite and schizont and then cytoadherence to cell differentiation 36 (CD36), intercellular cell adhesion molecule-1 (ICAM-1), and vascular cell adhesion molecule (V-CAM) and E-selectin were examined. The relative binding sizes of parasite subpopulations to human receptors were measured by mini-column cytoadherence method. The adhesion phenotype of *P. falciparum*-A4 line was evaluated by in vitro static, flow-based and mini-column binding assays.

**Results:**

The relative binding size of ITG, A4 and 3D7 clones to a column made with CHO/ICAM-1 was 68%, 54% and 0%, respectively. The relative binding sizes of these lines to CHO/CD36 were 59.7%, 28.7% and 0%, respectively. Different field isolates had variable sizes of respective CD36 and ICAM1-binding subpopulations. A4 line had five different subpopulations each with different binding sizes.

**Conclusion:**

This study provided further evidence that *P. falciparum* isolates have different binding subpopulations sizes in an infection. Furthermore, measurement of ICAM-1 or CD36 binding subpopulations may practical to study the cytoadherence phenotypes of *P. falciparum* field isolates at the molecular level.

## Introduction

A distinctive characteristic of *Plasmodium falciparum* is the ability of the infected erythrocytes (IE) to bind to host vascular endothelial cells, infected and uninfected erythrocytes and platelets (1–3). This ability (cytoadherence) leads to the occlusion of the microvasculature in various tissues and organs, such as brain in cerebral malaria (4), therefore contributing directly to the pathogenesis of severe malaria disease (5). Cytoadherence of erythrocytes infected with *P. falciparum* is associated with the expression of *P. falciparum* erythrocyte membrane protein-1 (*Pf*EMP-1), a high molecular weight protein, highly variable, which is encoded by genes of the *var* family (6–7).

*Plasmodium falciparum* uses a variety of endothelial receptors for cytoadherence which have been recognized and described in details including cell differentiation 36 (CD36) (8–9), intercellular cell adhesion molecule-1 (ICAM-1) (10–11), and vascular cell adhesion molecule (V-CAM), endothelial leukocyte adhesion molecule (ELAM-1)(12). Previous studies established that *P. falciparum* isolates had different binding characteristics to endothelial receptors (13–15). For example, *P. falciparum* isolates divided to low- and high-ICAM-1-avidity binders (13) or some laboratory isolates had a range of binding capability to purified receptors (ICAM-1 and CD36) and endothelial cells (HUVEC and HDMEC) under both static and flow conditions (14). The molecular basis for this difference is not well understood but might be due to variation in the binding sites of major receptors (16); in addition to differences in the display and copy number of parasite adhesions on the surface of the IE (17) and the differences in the length of parasite ligand (*Pf*EMP1 protein) (14, 18–19). On the other hand, other studies indicated that more than one parasite lines with different binding characteristics present in any parasite population, whether taken directly from a patient or adapted for long-term in vitro cultivation (20–22). A recent study consider for a competition between IE for binding to endothelial clearly showed that *P. falciparum* variants compete for adhesion to endothelial receptors based on their efficiency of binding. It also suggested that variants from a mixed infection do not display uniform cytoadherence and therefore may differ in their ability to cause sickness (23).

In our previous work a new cytoadherence method was introduced which able to measure relative binding size of a parasite line to a particular endothelial receptor (24).

This study aimed to compare the relative binding size of three well defined laboratory parasite lines and four field isolates to CHO cells expressing ICAM-1, CD36 and CHO non- transfected cells. In addition, the relative size of binding subpopulations of A4 line to several receptors including CD36, ICAM-1, E-selectin, V-CAM was measured.

## Materials and Methods

### Parasites and cells

Three *P. falciparum* lines A4 (16–17), 3D7 (from NF54 from Netherland received from D. Walliker), ITG (9, 17) and four field isolates from Malawi were used. All parasites were cultured in human blood group O ^+^ using RPMI-1640 containing AB^+^ human serum (RPMI-HS) mostly described by Mphande et al.,2008 (25). Chinese Hamster Ovary cells (CHO) or CHO transfected with CD36 or with other receptors were cultivated as described by Vogt, 2008 (26). These cells were prepared by Dr. Russell Howard.

### Cytoadherence on mini-column

Mini-column adhesion assay and selection of a particular binding subpopulation of *P. falciparum* were performed as previously described (24). Briefly, a column was made by suspending Cytodex beads, previously covered with CHO cells, in a 1 ml pipette tip fitted with a polyethylene disc to retain the beads in the column. The column was then washed once with RPMI-1640 containing Fetal calf serum (RPMI-FCS) followed by the addition of 1 ml of *P. falciparum* culture at 2% hematocrit (all isolates used after about 30-36 hours retrieval at any parasitemia). The column was washed three times with RPMI-HS to remove unbound infected erythrocytes. Parasitemia was measured before and after passing the *P. falciparum-*infected erythrocytes through the column by counting at least 2,000 cells and the percent of retained infected erythrocytes was obtained. Each experiment was repeated at least three times.

### Cytoadherence under static conditions

The technique was carried out based on a method described by Marsh et al., 1988 (27). Parasites were at late trophozoite and schizont stages at 1% parasitemia and 5% hematocrit. The method was performed in two independent experiments and duplicate.

### Cytoadherence under flow conditions

The assay was performed on the base of the method carried out by Gray et al., 2003, using purified ICAM-1 (14). Parasites were at late trophozoite and schizont stages at ∼2% parasitemia and 1% hematocrit. The method carried out in two independent experiments and triple.

### Statistical analysis

Student's paired t-test test was used to measure differences in parasitemia before and after passing through different min-column. ANOVA and Kruskal-Wallis tests were used to compare the mean of adhesion rate of A4 line to various CHO cells. T-test was used to compare the mean of adhesion rate of parental A4 line and ICAM-1 binding subpopulation of A4 to purified ICAM-1 under flow conditions. Calculations were performed using the SPSS 18.0 Software.

## Results

### Mini-column cytoadherence

**Fig. 1:**
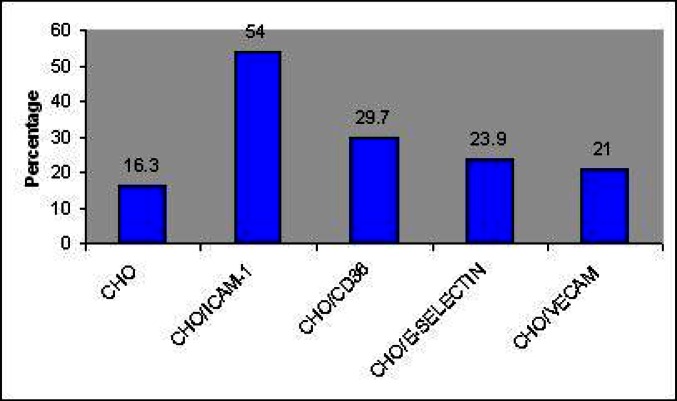
Adhesion of A4 to CHO transfected and non- transfected cells with mini-column binding assay. Data is shown as mean of the percentage of retained infected erythrocyte which was calculated from parasitemia before and after passing through different mini-column. Differences between the adhesion rate of A4 line to various CHO cells, each expressing a unique endothelial receptor, were statistically significant (*P*=0.000)

With the exception of 3D7, all parasite lines showed some binding to a column made with CHO/ICAM-1 cells (*P*<0.05). The relative sizes of ICAM-1-subpopulations varied from 0 (3D7) to 68% (ITG) among the tested isolates. The same pattern was seen with CHO cells expressing CD36 and the relative CD36-binding subpopulations size was from 0 (3D7) to 59.7% (ITG). The relative sizes of binding subpopulations to CHO cells alone ranged from 16.3% to 18.4% (Table 1). The field isolates had similar binding subpopulation size, ranging from 38% (e.g. 340 to ICAM-1) to 48% (6 to ICAM-1) (Table 1). The population of infected erythrocytes bound to the column in terms of the percentage of cells retained on the columns is also shown in Table 1. However, erythrocytes infected with *P. falciparum* have been reported to bind a number of host receptors (28), but most reports have been tested to one or two receptors with classical adhesion assays. Here, CHO transfected cells which expressing only one putative endothelial receptor were used to determine the relative size of various binding subpopulations of A4 line by the mini-column technique. Results obtained here showed that A4 parasites are able to bind to different receptors and therefore consist of at least five variant subpopulations. Each varies in size ranging from 54% to 16.3% for CHO/ICAM-1 and CHO-cells, respectively (Fig. 1).

**Table 1:** Adhesion of ITG, A4, 3D7 and four field isolates (340, 174, 6 and 350) from Malawi to CHO transfected and non- transfected cells with mini-column binding assay. Data is shown as mean of percentage of parasitemia before (P.B), after (P.A) and retained infected erythrocytes (R.IE). The binding assay to CHO cell was preformed for ITG, A4 and 6. Differences between the mean of parasitemia before and after passing through the mini-column for all isolates, except 3D7, were statistically significant (*P*<0.05)

Isolates Receptors	A4 P.B	P.A	R.IE	ITG P.B	P.A	R.IE	3D7 P.B	P.A	R.IE	340 P.B	P.A	R.IE	174 P.B	P.A	R.IE	6 P.B	P.A	P.A R.IE	350 P.B	P.A
ICAM-1	1.80 41	.83	54	.85	.27	68	.73	.87	0	.82	.51	38	1.11	.6	46	1.33	0.69	48	2	1.18
CD36	1.29 34	.92	28.7	.77	.31	59.7	.78	.78	0	1.12	.6	46	1.08	.58	46	1.9	1.04	45.	2	1.32
CHO	1.34	1.12	16.3	1.47	1.20	18.4	–	–	–	–	–	–	–	–	–	2.6	2.15	17.3	–	–

The ICAM-1 binding subpopulation of A4 line which previously selected by the mini-column assay was used as a control when the method was performed for CHO/ E-selectin. Results obtained from this experiment showed that the ICAM-1 selected subpopulation of A4 was not able to bind to E-selectin while about 24% of parent population was retained in the column.

### Cytoadherence assay under static conditions

**Fig. 2:**
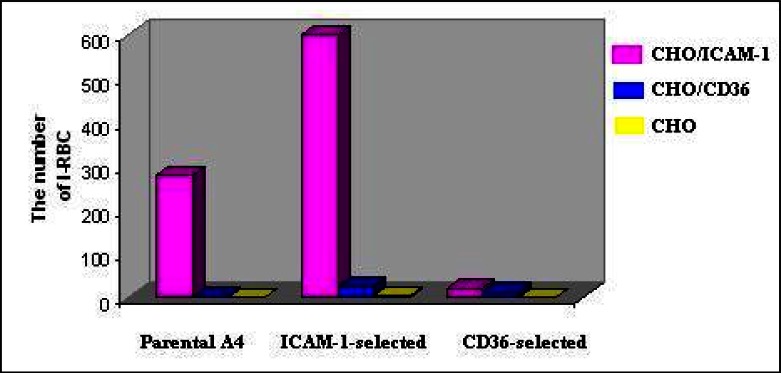
Adhesion of A4 line, ICAM-1 and CD36 selected subpopulations of A4 to CHO cells expressing ICAM-1, CD36 and parental CHO cells under static conditions. These subpopulations were selected by mini-column technique. Data is shown as mean number of bound IE of each population to different CHO cells. Differences between the mean number of bound IE of parental population line and different binding subpopulations of A4 to various CHO cells, each expressing a unique endothelial receptor, were statistically significant (*P*=0.000)

Binding assay on cover-slips with A4 line indicated that the number of the IE-A4 bound to various CHO cells was low and the parasite bound more to CHO/ICAM-1 in comparison to CHO/CD36 or CHO cells. The mean number of IE bound to ICAM-1 was 280 for ∼500 CHO/ICAM-1 cells while the mean number of bound IE to ∼500 CHO/CD36 and CHO cells were 1 and 0, respectively. This experiment was repeated with ICAM-1 and CD36- selected subpopulation of A4 (24) in order to identify whether the selection of parasites using the on-column technique could increase the number of bound infected cells to a particular receptor. Results showed that the mean number of ICAM-1-selected parasite bound to CHO/ICAM-1 (∼500 cells) increased to 600. It also indicated that the selected subpopulation was able to bind more to CD36 comparing with unselected parasites. The same pattern was seen when CD36-selected parasite used to perform adhesion assay on cover- slips (Fig. 2). Furthermore, the mean number of CD36-selected subpopulation bound to ICAM-1 was significantly low in comparison with ICAM-1-selected line and unselected population (Fig. 2).

### Cytoadherence under flow conditions

Cytoadherence under flow conditions using unselected and ICAM-1 selected of A4 line was performed in order to compare the adherence ability of the parasites under static conditions and to characterise further the on-column cytoadherence technique.

**Fig. 3:**
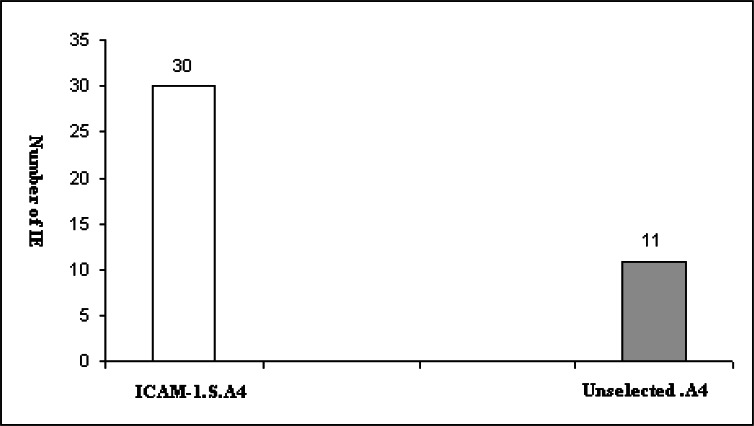
Adhesion of A4 line and ICAM-1 selected subpopulation of A4 line to purified ICAM-1 under flow conditions. The ICAM-1 binding subpopulation was selected by mini-column technique. Data is shown as mean ratio of binding of each population of the parasite. Difference between the mean rate of binding of parental population line and ICAM-1 binding subpopulations of A4 to purified ICAM-1 was statistically significant (*P*=0.007)

Fig. 3 shows the results obtained from counting the number of infected cells bound to purify ICAM-1. The mean number of bound infected cells of the ICAM-1 selected subpopulation and unselected parasites to this protein were 30 and 11 per mm^2^, respectively.

## Discussion

Infected erythrocytes with late trophozoites and schizonts of *P. falciparum* do not appear in peripheral blood but they are sequestered in deep tissues. The mechanism of sequestration of erythrocytes infected with late stage parasites has been widely studied, and a number of in vitro models and techniques of cytoadherence have been developed. In our previous study, we developed a new cytoadherence method which enabled the quantitative study of subpopulations of *P. falciparum* within an infection (24). Here, the relative size of ICAM-1 and CD36 binding subpopulations of seven parasite lines were measured by mini-column technique. This study also investigated the adhesion properties and compare binding ability to several endothelial receptors using mini-column, *in vitro* static and flow-based adhesion assays. Many studies on binding using *in vitro* static and flow-based adhesion assays have shown that CD36 and ICAM-1 binding subpopulations are common adhesion feature in the parasite population (10, 29–31). These studies also indicated that the level of adhesion among infected erythrocytes with different isolates of *P. falciparum* (10) or between isogenic parasites (31) varied widely. Moreover, ITG is a strong ICAM-1 binder that also adheres to CD36 (10). Our findings were in agreement with the results obtained by others as demonstrated all lines, except the 3D7 clone, were capable of binding to CHO/ICAM-1 or CHO/CD36 or different isolates have shown variable ICAM-1 and CD36-binding subpopulations ranging from 0 to 68% and 0 to 59.7%, respectively. Furthermore, all isolates except one of field isolates (number 340) bound better to ICAM-1 than to CD36 and ITG isolate had the largest ICAM-1 binding subpopulation. These results are in agreement with previous data showing that ICAM-1 could be the critical molecule for adhesion of *P. falciparum* to endothelial cells (14) (Table 1). But it is in contrast with results obtained from Udomsangpetch and co-workers which showed that the adherence of sixty field isolates to CD36 was at least 10-fold higher than other receptors (32).

On the other hand, previous studies have revealed that each isolate of *P. falciparum*, whether taken directly from patients or adapted to long term cultivation, contains a variety of subpopulations (21, 22). This finding confirmed by the current study as shown each tested isolate has at least three variant subpopulations with distinct cytoadherence phenotypes (binding to ICAM-1, binding to CD36 and binding to CHO). Two further transfected CHO cells (CHO/V-CAM and CHO/E-selectin) used to perform min-column adhesion assay for A4 line and therefore, this clone has five variant subpopulations (binding to V-CAM and to E-selectin). The binding subpopulations size of A4 clone for CHO/ICAM-1, CHO/CD36, CHO/E-selectin, CHO/V-CAM and CHO cells were 51%, 31.5%, 24%, 21% and16.4%, respectively. These results indicate that there is a small subpopulation of the A4 isolate which is able to bind to V-CAM, E-selectin and CHO cells. This finding may have three possible explanations: (i) interaction between ligand and receptor is weak, (ii) there is a rare *var* gene product in the total population of A4 which is able to bind to these molecules or (iii) another molecule may play a role as adhesive ligand of the IE to these receptors. These results are supported by the results obtained from another study which showed that the rolling velocity on V-CAM was 5 to 10-fold higher than on CD36 and ICAM-1(33). Also, a recent study revealed that very rare *Pf*EMP1 involved in adhesion properties to several receptors such as VCAM and E-selectin or it may only play an additive role in overall binding affinity (31). In addition results obtained from min-column binding assay with CHO/E-selectin and ICAM-1 selected subpopulation of A4 showed that the selected parasite did not bind to the E-selectin and supported the hypothesis that a rare *Pf*EMP-1 can be involved in adhesion to this receptor which is removed by selection process. Further adhesion assays with CHO/V-CAM or E-selectin using various binding subpopulations of the A4 parasite would be necessary to confirm this explanation.

Binding assays in static conditions using the unselected, ICAM-1 and CD36-selected subpopulations of A4 showed that all clones have a high affinity for adherence to ICAM-1 and bound more to CHO/ICAM-1. This could have two possible explanations: (i) the A4 isolate is a clone derived from ITG strain on the basis of its binding to ICAM-1 and (ii) the selection of parasites based on binding to ICAM-1 may result in a restricted subset of antigenic variants while the selection of parasites adherent to CD36 may be a characteristic of the majority of *P****. falciparurn* variant antigenic types. This would be in agreement with observations in other studies which indicated that field isolates adhered more frequently to CD36 than to ICAM-1 (10, 32, 34). Therefore, selection of parasites on the basis of binding to CD36 may lead to the concentration of parasites with various adhesive phenotypes and therefore able to bind to ICAM-1. It is in contrast with results obtained from a study that indicated that filed isolates selected on CD36 adhered almost exclusively to CD36 whereas, 80% to 90% of IE selected on ICAM-1 could also adhere to CD36 (32). The possible explanation for the differences were seen here is that the A4 clone is a parasite line which was derived from a strain on the basis of its binding to ICAM-1.

Also, the number of infected erythrocytes bound to CD36 using ICAM-1 selected parasites was increased in comparison to the parent population. The presence of a particular subpopulation within selected parasites which are able bind to two receptors at the same times could be a possible explanation. This is supported by the results obtained from other studies which indicated the percentage of cytoadherence of A4 to endothelial cells which was reduced when monoclonal antibodies against CD36 and ICAM-1 were used at the same time (22, 35). Also, a more recent study indicated that one selected parasite sub-line simultaneously expressed two different *var* genes as surface antigens, on single IE and it is related to adhesion and malaria pathogenesis (36). The binding assays under flow condition using purified proteins showed that the selection of the parasite with mini-column adhesion assay led to increase the number of IE bound to ICAM-1 (Fig. 3). Also, the number of IE bound to ICAM-1 was greater under flow conditions in comparison to static conditions. These results are in agreement with other studies which indicated that cytoadherence of infected red blood cells to endothelial receptors under flow conditions are more realistic (37–39).

## Conclusion

This study provided further evidence that *P. falciparum* infections are composed of multiple subpopulations based on cytoadherence characteristic. It also demonstrated that various *P. falciparum* isolates have different binding subpopulations sizes. Measurement of ICAM-1 or CD36 binding subpopulations size may useful to evaluate the cytoadherence phenotypes of *P. falciparum* field isolates at the molecular level.
